# A slip law for hard-bedded glaciers derived from observed bed topography

**DOI:** 10.1126/sciadv.abe7798

**Published:** 2021-05-14

**Authors:** Christian Helanow, Neal R. Iverson, Jacob B. Woodard, Lucas K. Zoet

**Affiliations:** 1 Department of Mathematics, Stockholm University, SE - 106 91 Stockholm, Sweden.; 2 Department of Geological and Atmospheric Sciences, Iowa State University, Ames, IA 50011, USA.; 3 Department of Geoscience, University of Wisconsin-Madison, Madison, WI 53706, USA.

## Abstract

Ice-sheet responses to climate warming and associated sea-level rise depend sensitively on the form of the slip law that relates drag at the beds of glaciers to their slip velocity and basal water pressure. Process-based models of glacier slip over idealized, hard (rigid) beds with water-filled cavities yield slip laws in which drag decreases with increasing slip velocity or water pressure (rate-weakening drag). We present results of a process-based, three-dimensional model of glacier slip applied to measured bed topographies. We find that consideration of actual glacier beds eliminates or makes insignificant rate-weakening drag, thereby uniting process-based models of slip with some ice-sheet model parameterizations. Computed slip laws have the same form as those indicated by experiments with ice dragged over deformable till, the other common bed condition. Thus, these results may point to a universal slip law that would simplify and improve estimations of glacier discharges to the oceans.

## Teaser

Numerical simulations of glacier slip over diverse hard beds indicate Coulomb slip behavior like that of deformable beds.

## INTRODUCTION

Major contributions of ice discharge to the oceans that result largely from rapid slip of glaciers over their beds are accelerating mass losses from ice sheets and associated sea-level rise (*1*, *2*). Both slow-moving (*3*) and fast-moving (*4*–*6*) parts of ice sheets can rest wholly or in part on hard beds that behave rigidly so that ice slips over them rather than deforming them. Where fast-flowing ice rests mostly on deformable till (“soft” beds), hard-bedded zones can have disproportionately large effects on modeled ice losses (*7*). Moreover, glacier slip over hard beds causes erosion of bedrock that modulates uplift rates in mountain belts (*8*) and rates of chemical weathering that alter climate (*9*).

Models of glacier flow in response to climate forcing (*2*, *10*) and of orogen-scale glacial erosion and its effects (*11*, *12*) therefore require a slip law for hard beds that relates drag at the bed, τ_b_, to slip velocity (*13*). Modeling studies indicate acute sensitivity of glacier response to the form of this relationship (*2*, *14*–*16*), which is affected by water-filled cavities that persist in the lees of bumps on the bed where ice pressure on the bed is low (*17*). The cavity water pressure, subtracted from the ice-overburden pressure, yields an effective pressure, *N*, that also contributes to the balance of forces on the bed. Quasi-static equilibrium requires that for two-dimensional (2D) beds, the ratio, τ_b_/*N*, cannot exceed the maximum slope, *m*_max_, of the up-glacier (stoss) sides of bed bumps (*18*).

**Fig. 1:**
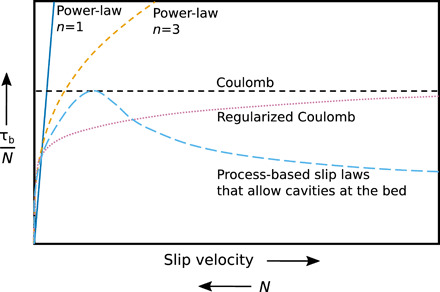
Slip laws for glaciers. Slip laws used in ice-sheet models (solid blue, dashed orange, and dotted purple) and a generalization of laws derived from process-based models of glacier slip that account for the effect of water-filled cavities at glacier beds (dashed light blue).

Process-based models for hard-bedded slip with cavities indicate slip laws with a common form: With increasing slip velocity or decreasing *N*, τ_b_/*N* increases, peaks at some fraction of *m*_max_, and then decreases at higher slip velocities (Fig. 1) (*19*–*23*). The decrease in τ_b_/*N*, called rate-weakening drag, is caused by cavity growth with increasing slip velocity that shifts shrinking zones of ice-bed contact toward the tops of bumps. Owing to bump convexity, this shift reduces slopes of stoss surfaces in contact with ice. Laboratory experiments confirm this drag relationship for a sinusoidal bed (*24*).

The potential consequences of this slip law are severe: Weather or climate changes that increase slip velocity or decrease *N* would decrease resistance to slip, promoting faster flow and glacier instability, including glacier surge propagation (*19*, *25*). Despite convergence of process-based theories on this slip law, nearly all large-scale models of glacier motion parameterize drag to increase monotonically with slip velocity, with varying degrees of nonlinearity bounded by Coulomb slip in which τ_b_/*N* is independent of slip velocity (Fig. 1) (*2*, *7*, *10*, *13*–*16*). A major concern in applying the rate-weakening drag indicated by process-based theories is that most of them consider only 2D beds (*19*, *20*, *22*, *23*) with regular geometries. In the only 3D model, rate-weakening drag was also found, but bed bumps were periodic and of uniform size and shape (*21*). Thus, the fundamental question of whether rate-weakening drag characterizes actual glacier beds is unanswered.

## RESULTS

### Glacier-bed topography

Processes of glacier slip occur within a few meters of where ice contacts bedrock (*26*). We thus consider scales of bedrock topography ranging from a few decimeters to tens of meters. Bedrock topography at this scale is determined by patterns of bedrock erosion by glaciers that depend on the lithology of the bed and its distribution and orientation of preglacial discontinuities, such as fractures, bedding planes, and foliation (*27*, *28*). Thus, any effort to bracket the range of slip behavior for hard glacier beds needs to consider their geological and hence morphological variability.

**Fig. 2:**
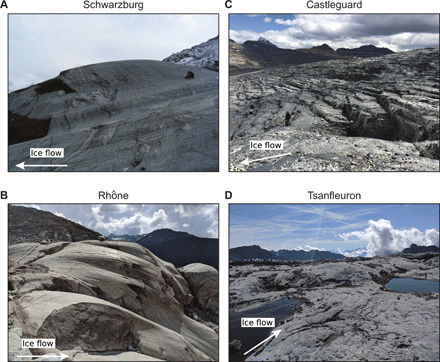
Photographs of bedrock from proglacial areas. (**A**) Schwarzburg, (**B**) Rhône, (**C**) Castleguard (photo by K. Williams), and (**D**) Tsanfleuron. Bedrock lithologies are granitic gneiss and granite, at Schwarzburg and Rhône glaciers, respectively, and limestone at Castleguard and Tsanfleuron glaciers. Ice flow directions in (A) to (D) are denoted by white arrows.

On bedrock surfaces recently exposed by glacier recession, we measured diverse topographies of former glacier beds at high resolution with either a terrestrial laser scanner (TLS) or photogrammetry from an unmanned aerial vehicle (UAV) (see Materials and Methods). Proglacial areas selected for study, in Alberta, Canada, and in Switzerland (see figs. S1 and S2), consist of well-exposed sedimentary, metamorphic, and igneous bedrock with contrasting patterns of discontinuities. Associated bed morphologies range from meter-scale steps with planar stoss treads trending sub-perpendicular to the glacier-slip direction to bumps with convex stoss surfaces streamlined obliquely to the slip direction (Fig. 2).

**Fig. 3:**
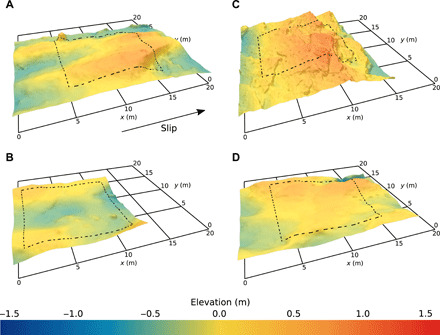
Representative topographies of bedrock surfaces exposed by glacier recession. REAs (outlined in dashed black, 100 m^2^) and surrounding topography used in the numerical model for the proglacial bedrock of (**A**) Schwarzburg Glacier, Switzerland; (**B**) Rhône Glacier, Switzerland; (**C**) Castleguard Glacier, Alberta, Canada; and (**D**) Tsanfleuron Glacier, Switzerland.

High-resolution surveys of proglacial bedrock span areas up to 0.7 km^2^, but because modeling glacier slip over 3D beds is computationally intensive, we develop sliding laws over smaller areas (140 to 270 m^2^). These areas enclose subsections of the bed, which we call representative elementary areas (REAs), chosen to be morphologically representative of the full areas surveyed. REAs are identified using nine morphological characteristics that potentially affect sliding physics, and principal component analysis (PCA) is used to score the importance of each characteristic (see fig. S3). Scores for the full areas surveyed are compared to those for subsections of the bed to isolate REAs (Fig. 3). The REA for a particular surveyed area does not reflect its full morphological variability but does contain its most characteristic elements. Distinct morphologic differences, which largely reflect contrasting patterns of bedrock discontinuities, are evident among the REAs of the surveyed areas (Fig. 3).

### Computed slip laws

**Fig. 4:**
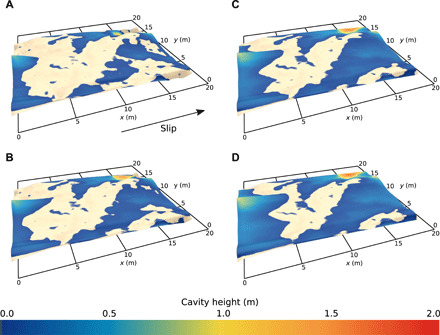
Cavity geometry and zones of ice-bed contact. Results of the numerical model from Tsanfleuron showing the geometry of the glacier sole for velocities *u*_e_ of (**A**) 10 m/a, (**B**) 20 m/a, (**C**) 50 m/a, and (**D**) 100 m/a. Zones of ice-bed contact (beige) are located on stoss sides of the bed topography. The color scale indicates the local heights of water-filled cavities.

To compute relationships between τ_b_/*N* and slip velocity for the bed topographies of the REAs, we numerically model 3D flow of pure ice at its pressure-melting temperature in a boundary layer at the bed much thicker than the topographic relief (see Materials and Methods) (*21*). Ice at the top of the layer is driven at a uniform speed down-glacier, as indicated by the average orientation of glacial striations on the bed, and cavities at the bed evolve to a steady size and shape (Fig. 4) under a steady, uniform effective pressure, *N*. Regelation is neglected. Owing to water that divides ice from rock, either in a thin film in zones of ice-bed contact or in cavities, local shear tractions on the sole of the ice layer are negligibly small. Therefore, the areally averaged drag at the bed, τ_b_, is the integral of components of normal stresses on the ice sole parallel to the mean slip direction. Computing the stress distribution numerically is complicated by the irregular topography of the REAs, which rules out assigning at their edges periodic boundary conditions like those used in numerical simulations of glacier slip over simpler beds (*20*, *21*). To circumvent this problem, slices of bed topography measured directly adjacent to REAs are included in the modeling domain, with this additional topography tapered systematically near the domain edges to allow application of periodic boundary conditions (see Materials and Methods and fig. S4). We compute τ_b_/*N* with a steady velocity applied to the top of the ice layer and consider increasingly large velocities until associated decreases in ice-bed contact area are very small (fig. S5). We determine the slip velocity, *u*_b_, in each case by integrating the velocity at the sole of the ice layer in the down-glacier direction. Resultant slip laws are presented as scaled, dimensionless drag relations (*19*–*21*, *23*) of the form

$$\frac{\tau_{b}}{N}=f(\frac{u_{b}}{N^{n}A_{s}})$$ (1)

where *n* is the stress exponent, taken to be 3.0, in the power-law constitutive rule for ice deformation (*29*). The constant *A*_s_ depends on the ice rheology and morphology of the bed and is defined on the basis of the long-standing drag relation for the case of no cavities (*30*)

$$\tau_{b}={\left(\frac{u_{b}}{A_{s}}\right)}^{1/n}$$ (2)

This scaling of slip velocity allows comparison among the REAs of differences in drag relations with and without cavities and results in a relation that is independent of whether the velocity or *N* is varied. Owing to the irregular morphologies of the REAs, which preclude scaling *A*_s_ to a simple geometric attribute of the bed, the value of *A*_s_ is computed through application of the model at velocities too small to induce cavity growth.

**Fig. 5:**
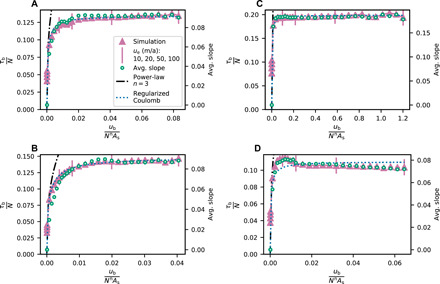
Computed slip laws. Slip laws for REAs of the former beds of (**A**) Schwarzburg Glacier, Switzerland; (**B**) Rhône Glacier, Switzerland; (**C**) Castleguard Glacier, Alberta, Canada; and (**D**) Tsanfleuron Glacier, Switzerland. Values of τ_b_/*N* are shown for a power-law slip law (*n* = 3) corresponding to no water-filled cavities at the bed (dash-dotted black line) and for the model simulations in which cavity formation at the bed was allowed (purple triangles). Also shown is the average slope of the bed in contact with ice (green circles) and a fit to the model simulations using the regularized Coulomb parameterization of Eq. 3, with fitted values *C* = 0.13 for Schwarzburg Glacier, *C* = 0.15 for Rhône Glacier, *C* = 0.20 for Castleguard Glacier, and *C* = 0.11 for Tsanfleuron Glacier (dotted blue line). Purple vertical bars mark model simulations for velocities *u*_e_ of 10, 20, 50, and 100 m/a, increasing from left to right (see Fig. 4). The slip laws are computed using a boundary-velocity range *u*_e_ = 0.1 to 100 m/a and *N* = 0.4 MPa (80% of *p*_i_).

Conspicuously lacking from the derived drag relations (Fig. 5) is the significant rate-weakening drag (Fig. 1) indicated by previous process-based models of hard-bedded glacier slip (*19*–*23*). For the REAs from the igneous and metamorphic beds surveyed, τ_b_/*N* increases toward a bounding value with increasing scaled slip velocity (Fig. 5, A and B). For a sedimentary bed consisting of quasiperiodic steps with nearly planar stoss surfaces and steep lee surfaces, the bounding value of τ_b_/*N* is reached at a very low slip velocity, so the drag relation closely approximates Coulomb behavior (Fig. 5C). The Coulomb behavior in this case is in contrast to mildly nonlinear relations between drag and slip velocity sometimes assumed for hard beds (*7*). A second sedimentary bed with more convex stoss surfaces indicates slight rate-weakening drag (Fig. 5D) but also approximates Coulomb behavior. Repeating these calculations for these two sedimentary proglacial areas using different REAs yields drag relations with similar forms (fig. S6), although in the case of the Castleguard proglacial area the magnitude of τ_b_/*N* is larger for the second REA because its up-glacier–facing bed slopes are steeper (fig. S6B). The general forms of the slip relations are not sensitive to domain-edge tapering (fig. S7).

Forms of the derived drag relations are controlled by the slopes of stoss surfaces in contact with ice (Fig. 5). Although the maximum stoss slope, *m*_max_, controls drag for the case of idealized beds (*18*, *23*), for the irregular, 3D bed topography considered here, the maximum stoss slope in contact with ice is locally severe but spans an inconsequentially small area. We find that the average bed slope, parallel to the mean flow direction, in contact with ice scales predictably with τ_b_/*N* as cavities get larger with increasing scaled slip velocity (Fig. 5). Rate-weakening drag, therefore, is not significant because stoss slopes of bumps in contact with ice tend to, with increasing slip velocity and cavity size, increase either slowly (Fig. 5, A and B) or rapidly (Fig. 5, C and D) toward limiting values rather than decrease. This effect can be due to stoss surfaces that are planar rather than convex (Figs. 2C and 3C). However, it arises more generally because actual 3D glacier beds are insufficiently periodic along a down-glacier flow line for cavities to commonly extend beyond the inflection points of convex stoss surfaces immediately downstream. This same lack of periodicity along a flow line also leaves some unusually steep stoss surfaces in contact with ice, which also inhibits rate-weakening drag (*22*).

Calculated magnitudes of τ_b_/*N* are less than 0.2 (Fig. 5), which is less than steady-state friction coefficients for tills [0.32 to 0.84 (*31*, *32*)], but basal drag is generally greater for hard beds than for soft beds (*31*). This observation highlights the importance of subglacial hydrology. By impeding water drainage, soft beds promote higher subglacial water pressure than hard beds and thus have sufficiently low values of *N* to cause lower slip resistance than hard beds (*17*).

## DISCUSSION

### Hard-bed parameterization

These results indicate that a process-based slip model applied to observed glacier beds does not commonly produce rate-weakening drag. This finding qualitatively aligns the small-scale physics of hard-bedded slip with slip parameterizations of ice-sheet models that, on heuristic grounds, neglect the possibility of rate-weakening drag.

Our results also support the hypothesis that when water-filled cavities persist at the bed, the adverse slopes of parts of bumps in contact with ice control τ_b_/*N* (Fig. 5) (*18*–*24*). This observation helps guide consideration of the effects on basal drag of bumps that are larger than the REAs and hence neglected in the modeling. Except near glacier margins where ice is thin, leeward cavities at scales greater than the REAs (i.e., tens of meters) will tend not to persist, owing to insufficient subglacial water discharge needed to sustain large, water-filled, pressurized cavities. Nevertheless, on the up-glacier–facing slopes of larger bumps (i.e., “hills”), superimposed smaller bumps of the scales modeled herein will result in steeper up-glacier–facing slopes than in the absence of such hills. Maximum up-glacier–facing bed slopes set the value of Iken’s bound (*18*) and *C* in Eq. 3 (*20*, *21*, *23*), so their values and values of τ_b_/*N* would likely be somewhat larger than we have calculated (Fig. 5) if larger proglacial areas could have been modeled. However, because cavities at the scales of hills are expected to be absent or rare, the form of the calculated slip laws, which results from cavity growth with increasing slip velocity, is likely to be insensitive to hills on the bed.

The slip parameterization most commonly applied in ice-sheet models is Eq. 2, which is rooted in the physics of hard-bedded sliding without water-filled cavities at the bed (*30*). It provides a poor estimate of drag for the case of *n* = 3 (Fig. 5). Much larger values of *n* are sometimes used in ice-sheet models that use this parameterization (*2*, *7*, *33*), but it is then fully detached from process-based models of sliding physics because *n* effectively becomes a free parameter rather than having a value that, through the constitutive rule for ice deformation, depends on the properties of ice. Schoof (*23*), on the other hand, neglected the rate-weakening indicated by his 2D, process-based slip model to heuristically suggest a parameterization in which τ_b_/*N* increases toward an upper bound.

To allow comparison with our calculated drag relations, we scale Schoof’s parameterization [regularized Coulomb law (*15*)] as

$$\frac{\tau_{b}}{N}=C{\left(\frac{u_{b}}{u_{b}+A_{s}C^{n}N^{n}}\right)}^{1/n}$$ (3)

where *C* depends on the bed morphology (*20*). Although *C* cannot be readily estimated from the irregular topographies of the REAs, slip models require that *C* < *m*_max_ (*20*, *23*). Using *C* as a fitting parameter with this constraint indicates that Eq. 3 can provide a good fit to our model results (Fig. 5).

### Universal slip law?

Our results indicate that Eq. 3 is a reasonable parameterization for slip of glaciers over actual hard beds. Although the details of relevant processes are different, a slip law of the same form for soft beds (*16*, *34*) is supported by results of recent laboratory experiments on till (*34*). Moreover, application to Pine Island Glacier, Antarctica, of this slip law indicates that it accounts for decadal velocity changes substantially better than unbounded slip laws (i.e., Eq. 2) (*15*). Among applications of unbounded slip laws, those with sufficient nonlinearity to more closely approximate the regularized Coulomb behavior of Eq. 3 commonly best optimize fits of flow model results to remote-sensing observations (*33*, *35*). Therefore, a slip law of the form of Eq. 3 may be universally applicable, allowing its parameters to be determined through application of observationally constrained glacier flow models without knowledge of the bed type (*13*). Given the sensitivity of modeled glacier discharges to various slip-law formulations, focusing on a single slip law and determination of its parameters would streamline efforts to estimate the contributions of glaciers to future sea-level rise.

## MATERIALS AND METHODS

### Digital elevation model data

Digital elevation models (DEMs) for the four proglacial areas are constructed using data from a TLS and photogrammetry techniques with images collected from a UAV (fig. S1). TLS data collected with a Riegl VA-400 scanner are used for the proglacial area of Castleguard Glacier. Scan positions are selected to assure scan overlap and mitigate shadow effects from bedrock features. The resulting point-cloud data are merged, filtered using reflectance (values ≤ − 25) and deviation (values ≤35) attributes, and manually cleared of spurious points with the RiSCAN Pro software package (*36*). For the three other proglacial areas (Schwarzburg, Rhône, and Tsanfleuron), photogrammetry techniques are applied to images collected from a UAV. The UAV is fitted with a 1-inch, 20-megapixel complementary metal oxide semiconductor camera that has an F2.8 wide-angle lens with a 9-mm focal length. The UAV adjusts its flight altitude to maintain a uniform distance from the ground while collecting images taken looking perpendicular to the ground surface. The images are used to generate point clouds with Agisoft photogrammetry software (*37*), which then are scaled and georeferenced with ground control points (GCPs) located across the surveyed areas. The locations of GCPs are measured with a real-time kinematic Altus Positioning System APS-3 GPS system [horizontal resolution: 0.006 m + 0.5 parts per million (ppm); vertical resolution: 0.01 m + 1 ppm]. The DEMs are created from the point clouds of the TLS and photogrammetry surveys using the natural neighbor interpolation method and have resolutions of 0.1, 0.1, 0.05, and 0.1 m and total errors of 1.12, 0.03, 0.01, and 0.09 m for Schwarzburg, Rhône, Castleguard, and Tsanfleuron, respectively. Schwarzburg’s total error is highest due to GPS failure partway through the photogrammetry survey.

### REA selection

To determine an REA for each proglacial area, morphologic attributes of 10 m × 10 m subsections of a DEM are compared with those of the entire proglacial area. The DEMs are trimmed to a square and then subdivided into sections using a sliding window with a 2-m step spacing. The subsections are detrended, and their morphologies are characterized after Leach (*38*) and Stout *et al.* (*39*) with the following nine parameters: root mean square (RMS) roughness, RMS slope, and RMS curvature to characterize the degree of surface roughness; texture aspect ratio to characterize the isotropy of the topography; length asymmetry and slope asymmetry to characterize the asymmetry of bumps in the direction of ice flow; skewness to characterize the symmetry of the surface elevation distribution; kurtosis to characterize the sharpness of bumps; and a 2D correlation coefficient, *r*, between the Fourier spectrum of the proglacial area and that of each subsection. A description of how these parameters are calculated is provided in the Supplementary Materials (text S1).

To find the REA of a proglacial area, PCA (*40*) is performed on the nine parameters to help identify a subsection that is representative of the DEM (see the Supplementary Materials for more details about PCA). In contrast to multidimensional distance methods (e.g., Euclidean, cosine, or Mahalanobis) that give equal weight to all parameters regardless of the variance they capture, the PCA method determines the parameter weights to capture the most variance of the proglacial morphology dataset. As the parameters that best describe the DEM morphologies are unknown beforehand, this approach allows the determination of the most effective variables (PCs) for describing the morphology and the percentage of the total variance captured by these variables. To evaluate the level of similarity between a subsection and the DEM, a representative value for the entire proglacial area, *X*_ν_, is designated for each parameter, where ν is the parameter index. For *r*, *X*_ν_ is set to one, which represents a perfect power spectrum match between a subsection and the entire DEM (see text S1). For all other parameters, *X*_ν_ is set to the mean of the parameter set. The *X*_ν_ values are normalized with the other parameter values to a mean of zero and variance of one before performing the PCA, owing to the different units of parameters. The PC scores (magnitude of each PC in the PC coordinate system) are estimated for the normalized parameter values of individual subsections, γ_ν,*i*_, and the representative parameter values of the entire proglacial area, γ_ν_, where *i* denotes the subsection index. The representative PC scores, γ_ν_, are subtracted from each subsection’s score, γ_ν,*i*_, resulting in a similarity index, θ_i_, for each subsection that is used to find the most representative subsection of the DEM

$$\theta_{i}=\gamma_{\nu ,i}-\gamma_{\nu }$$ (4)

Here, values closer to zero reflect a greater degree of similarity between a given subsection and the entire proglacial area. Owing to the use of PCs, this similarity index intrinsically incorporates the appropriate weights of each parameter to best describe the surface attributes. Following Cattell and Vogelmann (*41*), the number of PCs used to locate an REA is determined by finding the point at which the percentage of the total variance captured by successive PCs converges to an approximately constant value and then including one PC beyond that point. For all of the proglacial areas, the subsections with the lowest combined θ value from the first two PCs are taken to best represent the morphology of the proglacial areas surveyed, as the percentages of the total variance of components beyond the first PC are relatively uniform. Loadings (level of correlation of each parameter with a PC) of the first two PCs for the four different DEMs are shown in fig. S3. The first two PCs account for 41, 42, 46, and 43% of the total variance for the proglacial areas of Schwarzburg, Rhône, Castleguard, and Tsanfleuron, respectively.

Application of periodic boundary conditions in the numerical model requires choosing a subsection with minimal elevation differences between its opposing sides. For each proglacial area, we consider the 50 subsections (∼0.5% of the total number of subsections of a proglacial area) with combined similarity indices closest to zero (greatest similarity) as a set of potential REAs. Each subsection of this set is expanded by 5 m in each direction using the surrounding bedrock topography, resulting in a 20 m × 20 m section centered on the statistically representative 10 m × 10 m subsection. As a measure of periodicity of any given rectangular section, we consider the *L*^2^-norm of the differences in elevation between opposing sides averaged over the length of each boundary. In an expanded section, this periodicity measure is calculated for every possible rectangular section centered on and containing the subsection. This procedure is performed for each expanded section in the set of potential REAs, with the rectangular section with the highest periodicity chosen as the section best suited for the numerical model. In cases where surface debris obscures bedrock, the rectangular section with the next highest periodicity is used. Last, a low-pass Butterworth filter of order six is applied to the selected rectangular section to remove wavelengths smaller than 0.25 m, making the shortest wavelengths present in the topographical data similar to the horizontal resolution of the numerical model.

To make the boundaries of a selected rectangular section fully periodic, we use a periodicizing tapering algorithm with a taper width of 1 m at the boundaries (see text S2). A 2D rectangular sliding window is used to average the elevation values within the tapered zone. The maximum size of the window is 2 m in each direction, with the window centered on the point (elevation value) that is averaged. The size of the window progressively decreases to zero as averaged coordinate points approach the inner edge of the tapered zone (see fig. S8). The varying size of the averaging window results in a smooth transition from the tapered part of the section to its untapered interior. In choosing a modeling domain, for the periodicity measure, we considered rectangular sections only large enough so that the centered REA is not affected by the tapering algorithm. For the four proglacial areas, comparing the PC scores of the tapered and low-pass–filtered rectangular section containing the selected REA to the corresponding unmodified section indicates that only a small statistical change in bed morphology is introduced by the tapering procedure. The relative change in the PC scores for the selected rectangular sections of the proglacial areas of Schwarzburg, Rhône, Castleguard, and Tsanfleuron are 0.07, 16.44, 1.31, and 0.98%, respectively. The high relative change of the Rhône proglacial area is due to the long wavelengths of its bed morphology and the small area of the selected rectangular section.

### Modeling strategy

The equations governing the viscous deformation of ice and steady-state geometry of the glacier sole, *z*_s_, are given by the Stokes equations (5) and kinematic free-surface equation (7). For the model, ice occupies a Cartesian domain in a boundary layer at the glacier bed, which consists of the rectangular domain that includes the REA and surrounding tapered regions. To the top of the domain, we apply a flow velocity, *u*_e_, and ice overburden pressure, *p*_i_. Gravity in the thin boundary layer can be neglected. At the bottom of the domain, the glacier sole either is in contact with the underlying bedrock, *z*_b_, or is the roof of a water-filled cavity, *z*_c_. The bedrock is taken to be impenetrable and the water pressure in cavities, *p*_w_, to be spatially uniform over the bed. Water in a thin film divides ice at its pressure-melting temperature from rock where water-filled cavities are absent, so the low viscosity of water results in negligible tangential stresses everywhere at the glacier sole (free slip). Basal melting and regelation are neglected. At the vertical boundaries of the tapered domain, periodic boundary conditions apply, so ice deforms during slip over morphologically repetitive bedrock of infinite extent.

For a particular bed and cavity geometry, the velocity, **u** = (*u_x_*, *u_y_*, *u_z_*), and pressure, *p*, are then given as the solution to

∇·σ=∇·τ−∇p=0 (balance of momentum) (5a)

∇·u=0 (conservation of mass) (5b)

$$n\cdot \sigma \cdot n=-p_{i},u_{x}=u_{e},u_{y}=0\;(\text{top}\;\text{boundary condition})$$ (5c)

$$n\cdot \sigma \cdot t=0\;\text{on}\;z_{s}\;(\text{free slip condition})$$ (5d)

$$u\cdot n=0\;\text{on}\;z_{b}\;(\text{impenetrability condition})$$ (5e)

$$n\cdot \sigma \cdot n=-p_{w}\;\text{on}\;z_{c}\;(\text{cavity roof condition})$$ (5f)

where σ and τ are the Cauchy and deviatoric stress tensors, and **n** and **t** are the outward-pointing normal and tangent to the boundary, respectively. The deviatoric stresses are related to strain rates through the constitutive equation for ice deformation (*29*)

$$\tau_{\text{ij}}=2\eta {\overset{\cdot }{\varepsilon }}_{\text{ij}}=B[{\overset{\cdot }{\varepsilon }}_{E}^{(1-n)/n}]{\overset{\cdot }{\varepsilon }}_{\text{ij}}$$ (6)

where η is the effective ice viscosity dependent on strain rate, with *n* = 3. In general, the rate factor *B* depends on temperature but is constant in the isothermal case that is appropriate here (*42*, *31*).

In Eq. 6, the strain-rate tensor and effective strain rate are given by ${\overset{\cdot }{\varepsilon }}_{\text{ij}}=\frac{1}{2}(\partial u_{i}/\partial x_{j}+\partial u_{j}/\partial x_{i})$ and ${\overset{\cdot }{\varepsilon }}_{E}=\frac{1}{\sqrt{2}}{\left({\overset{\cdot }{\varepsilon }}_{\text{ij}}{\overset{\cdot }{\varepsilon }}_{\text{ij}}\right)}^{1/2}$, respectively. The Stokes equations (5) describe steady flow, but steady-state geometries of cavities at the bed are not known a priori. Thus, a separate equation is needed to simulate the transient evolution of *z*_s_ to the desired steady state in which water-filled cavities at the glacier sole are not shrinking or expanding. Considering the glacier sole to be a free surface, with the additional condition that it be at or above the underlying bed at every point, the kinematic free-surface equation is

$$\partial_{t}z_{s}+u_{x}\partial_{x}z_{s}+u_{y}\partial_{y}z_{s}=u_{z},\text{such that}\;z_{s}\geq z_{b}$$ (7)

For a steady-state ice geometry, τ_b_ is the bed-averaged viscous drag in the general flow direction (*19*, *22*, *23*), calculated as

$$\tau_{b}=-\frac{1}{\Omega }\int_{z_{s}}(n\cdot \sigma \cdot n)n_{x}\mathrm{ds}$$

where Ω is the area of the computational domain in the horizontal plane and *n_x_* is the *x* component of **n**. In a corresponding manner, the average slip velocity in the direction of flow is calculated as

$$u_{b}=\frac{1}{\Omega }\int_{z_{s}}u_{x}\mathrm{dx}$$

The set of Eqs. 5 and 7 is solved numerically using the finite element software Elmer/Ice (*43*). All simulations are performed using nodal finite elements on meshes generated by extruding an unstructured triangulated (*x*/*y* plane) base mesh in 30 layers in the vertical (*z*) direction, resulting in prismatic/wedge elements. The same vertical mesh extrusion is used for all glacier topographies, with the density of element layers highest near the base of the computational domain. The top of the domain is fixed at *z* = 15 m, whereas the bottom of the domain adjusts to the geometry of *z*_s_ in each time step. All base meshes are generated with a characteristic cell size (triangle edge length) of 0.15 m using the frontal Delaunay algorithm through the software Gmsh (*44*). Owing to the different horizontal extents of the computational domains, this results in meshes consisting of 938,640 (Castleguard), 518,520 (Rhône), 962,460 (Schwarzburg), and 777,840 (Tsanfleuron) cells. Additional description of the numerical model is given by Helanow *et al.* (*21*) and its supporting information, where results of sensitivity and parameter tests can also be found.

## SUPPLEMENTARY MATERIALS

Supplementary material for this article is available at http://advances.sciencemag.org/cgi/content/full/7/20/eabe7798/DC1

## References

[1] The IMBIE Team, Mass balance of the Greenland Ice Sheet from 1992 to 2018. Nature 579, 233–239 (2020). 31822019 10.1038/s41586-019-1855-2

[2] C. Ritz, T. L. Edwards, G. Durand, A. J. Payne, V. Peyaud, R. C. A. Hindmarsh, Potential sea-level rise from Antarctic ice-sheet instability constrained by observations. Nature 528, 115–118 (2015). 26580020 10.1038/nature16147

[3] N. Maier, N. Humphrey, J. Harper, T. Meierbachtol, Sliding dominates slow-flowing margin regions, Greenland Ice Sheet. Sci. Adv. 5, eaaw5406 (2019). 31309154 10.1126/sciadv.aaw5406 PMC6620096

[4] T. Bradwell, D. Small, D. Fabel, R. K. Smedley, C. D. Clark, M. H. Saher, S. L. Callard, R. C. Chiverrell, D. Dove, S. G. Moreton, D. H. Roberts, G. A. T. Duller, C. Ó. Cofaigh, Ice-stream demise dynamically conditioned by trough shape and bed strength. Sci. Adv. 5, eaau1380 (2019). 31058217 10.1126/sciadv.aau1380 PMC6498188

[5] M. Krabbendam, N. Eyles, N. Putkinen, T. Bradwell, L. Arbelaez-Moreno, Streamlined hard beds formed by palaeo-ice streams: A review. Sediment. Geol. 338, 24–50 (2016).

[6] A. Muto, S. Anandakrishnan, R. B. Alley, H. J. Horgan, B. R. Parizek, S. Koellner, K. Christianson, N. Holschuh, Relating bed character and subglacial morphology using seismic data from Thwaites Glacier, West Antarctica. Earth Planet. Sci. Lett. 507, 199–206 (2019).

[7] S. Koellner, B. R. Parizek, R. B. Alley, A. Muto, N. Holschuh, The impact of spatially-variable basal properties on outlet glacier flow. Earth Planet. Sci. Lett. 515, 200–208 (2019).

[8] J. D. Pelletier, Glacial erosion and mountain building. Geology 36, 591–592 (2008).

[9] M. A. Torres, N. Moosdorf, J. Hartmann, J. F. Adkins, A. J. West, Glacial weathering, sulfide oxidation, and global carbon cycle feedbacks. Proc. Natl. Acad. Sci. U.S.A. 114, 8716–8721 (2017). 28760954 10.1073/pnas.1702953114 PMC5565423

[10] R. A. Bindschadler, S. Nowicki, A. Abe-Ouchi, A. Aschwanden, H. Choi, J. Fastook, G. Granzow, R. Greve, G. Gutowski, U. Herzfeld, C. Jackson, J. Johnson, C. Khroulev, A. Levermann, W. H. Lipscomb, M. A. Martin, M. Morlighem, B. R. Parizek, D. Pollard, S. F. Price, D. Ren, F. Saito, T. Sato, H. Seddik, H. Seroussi, K. Takahashi, R. Walker, W. L. Wang, Ice-sheet model sensitivities to environmental forcing and their use in projecting future sea level (the SeaRISE project). J. Glaciol. 59, 195–224 (2013).

[11] D. L. Egholm, S. B. Nielsen, V. K. Pedersen, J.-E. Lesemann, Glacial effects limiting mountain height. Nature 460, 884–887 (2009). 19675651 10.1038/nature08263

[12] D. Egholm, J. D. Jansen, C. F. Brædstrup, V. K. Pedersen, J. L. Andersen, S. V. Ugelvig, N. K. Larsen, M. F. Knudsen, Formation of plateau landscapes on glaciated continental margins. Nat. Geosci. 10, 592–597 (2017).

[13] B. Minchew, I. Joughin, Toward a universal glacier slip law. Science 368, 29–30 (2020). 32241936 10.1126/science.abb3566

[14] J. Brondex, O. Gagliardini, F. Gillet-Chaulet, G. Durand, Sensitivity of grounding line dynamics to the choice of the friction law. J. Glaciol. 63, 854–866 (2017).

[15] I. Joughin, B. E. Smith, C. G. Schoof, Regularized Coulomb friction laws for ice sheet sliding: Application to Pine Island Glacier, Antarctica. Geophys. Res. Lett. 46, 4764–4771 (2019). 31244498 10.1029/2019GL082526 PMC6582595

[16] V. C. Tsai, A. L. Stewart, A. F. Thompson, Marine ice-sheet profiles and stability under Coulomb basal conditions. J. Glaciol. 61, 205–215 (2015).

[17] A. G. Fountain, J. S. Walder, Water flow through temperate glaciers. Rev. Geophys. 36, 299–328 (1998).

[18] A. Iken, The effect of the subglacial water pressure on the sliding velocity of a glacier in an idealized numerical model. J. Glaciol. 27, 407–421 (1981).

[19] A. C. Fowler, Sliding with cavity formation. J. Glaciol. 33, 255–267 (1987).

[20] O. Gagliardini, D. Cohen, P. Råback, T. Zwinger, Finite-element modeling of subglacial cavities and related friction law. J. Geophys. Res. Earth Surface 112, F02027 (2007).

[21] C. Helanow, N. R. Iverson, L. K. Zoet, O. Gagliardini, Sliding relations for glacier slip with cavities over three-dimensional beds. Geophys. Res. Lett. 47, e2019GL084924 (2020).

[22] L. Lliboutry, General theory of subglacial cavitation and sliding of temperate glaciers. J. Glaciol. 7, 21–58 (1968).

[23] C. Schoof, The effect of cavitation on glacier sliding. Proc. R. Soc. A 461, 609–627 (2005).

[24] L. K. Zoet, N. R. Iverson, Experimental determination of a double-valued drag relationship for glacier sliding. J. Glaciol. 61, 1–7 (2015).

[25] K. Thøgersen, A. Gilbert, T. Schuler, A. Malthe-Sørenssen, Rate-and-state friction explains glacier surge propagation. Nat. Commun. 10, 2823 (2019). 31249287 10.1038/s41467-019-10506-4 PMC6597537

[26] G. K. C. Clarke, Subglacial processes. Annu. Rev. Earth Planet. Sci. 33, 247–276 (2005).

[27] M. Dühnforth, R. S. Anderson, D. Ward, G. M. Stock, Bedrock fracture control of glacial erosion processes and rates. Geology 38, 423–426 (2010).

[28] T. S. Hooyer, D. Cohen, N. R. Iverson, Control of glacial quarrying by bedrock joints. Geomorphology 153-154, 91–101 (2012).

[29] J. W. Glen, The creep of polycrystalline ice. Proc. R. Soc. A 228, 519–538 (1955).

[30] J. Weertman, On the sliding of glaciers. J. Glaciol. 3, 33–38 (1957).

[31] K. M. Cuffey, W. S. B. Paterson. *The Physics of Glaciers* (Academic Press, 2010).

[32] N. R. Iverson, Shear resistance and continuity of subglacial till. J. Glaciol. 56, 1104–1114 (2011).

[33] F. Gillet-Chaulet, G. Durand, O. Gagliardini, C. Mosbeux, J. Mouginot, F. Rémy, C. Ritz, Assimilation of surface velocities acquired between 1996 and 2010 to constrain the form of the basal friction law under Pine Island Glacier. Geophys. Res. Lett. 43, 10,311–10,321 (2016).

[34] L. K. Zoet, N. R. Iverson, A slip law for glaciers on deformable beds. Science 368, 76–78 (2020). 32241945 10.1126/science.aaz1183

[35] B. Minchew, M. Simons, H. Björnsson, F. Pálsson, M. Morlighem, H. Seroussi, E. Larour, S. Hensley, Plastic bed beneath Hofsjökull Ice Cap, central Iceland, and the sensitivity of ice flow to surface meltwater flux. J. Glaciol. 62, 147–158 (2016).

[36] Riegl Laser Measurement System (RiSCAN PRO, 2013).

[37] Agisoft LLC (Agisoft Photoscan Pro, 2017).

[38] R. Leach, *Characterisation of Areal Surface Texture* (Springer, 2013).

[39] K. Stout, L. Blunt, W. Dong, E. Mainsah, N. Luo, T. Mathia, P. Sullivan, H. Zahouani, *Development of Methods for Characterisation of Roughness in Three Dimensions* (Butterworth-Heinemann—Elsevier, 2000).

[40] J. Davis, *Statistics and Data Analysis in Geology* (Wiley, 2002).

[41] R. B. Cattell, S. Vogelmann, A comprehensive trial of the scree and kg criteria for determining the number of factors. Multivar. Behav. Res. 12, 289–325 (1977). 10.1207/s15327906mbr1203_2 26804294

[42] R. L. Hooke, *Principles of Glacier Mechanics* (Cambridge Univ. Press, ed. 2, 2005).

[43] O. Gagliardini, T. Zwinger, F. Gillet-Chaulet, G. Durand, L. Favier, B. De Fleurian, R. Greve, M. Malinen, C. Martín, P. Råback, J. Ruokolainen, M. Sacchettini, M. Schäfer, H. Seddik, J. Thies, Capabilities and performance of Elmer/Ice, a new generation ice-sheet model. Geosci. Model Dev. 6, 1299–1318 (2013).

[44] C. Geuzaine, J.-F. Remacle, Gmsh: A 3-D finite element mesh generator with built-in pre- and post-processing facilities. Int. J. Numer. Meth. Eng. 79, 1309–1331 (2009).

[45] J. T. Perron, J. W. Kirchner, W. E. Dietrich, Spectral signatures of characteristic spatial scales and nonfractal structure in landscapes. J. Geophys. Res. Earth Surface 113, F04003 (2008).

[46] Esri (World imagery, 2009); http://arcgis.com/home/item.html?id=10df2279f9684e4a9f6a7f08febac2a9.

